# Inhibiting CCN1 blocks AML cell growth by disrupting the MEK/ERK pathway

**DOI:** 10.1186/s12935-014-0074-z

**Published:** 2014-08-16

**Authors:** Chang-Chun Niu, Chen Zhao, Zhong Yang, Xiao-Li Zhang, Jing Pan, Chen Zhao, Wei-Ke Si

**Affiliations:** 1 Department of Clinical Hematology, Southwest Hospital, Third Military Medical University, Chongqing 400038, China; 2 Department of Clinical Laboratory, The Third People’s Hospital of Chongqing, Chongqing 400014, China; 3 The First Affiliated Hospital, Chongqing Medical University, Chongqing 400042, China

**Keywords:** CCN1, AML, ERK

## Abstract

**Background:**

CCN1 plays distinct roles in various tumor types, but little is known regarding the role of CCN1 in leukemia.

**Methods:**

We analyzed CCN1 protein expression in leukemia cell lines and in AML bone marrow samples. We also evaluated the effects of antibody- or siRNA-mediated inhibition of CCN1 on the growth of two AML cell lines (U937 and Kasumi-1 cells) and on the MEK/ERK pathway, β-catenin and other related genes.

**Results:**

U937 and Kasumi-1 cells had markedly higher CCN1 expression than the 5 other leukemia cell lines, and CCN1 protein expression was higher in the AML bone marrow samples than in the normal bone marrow samples. Blocking CCN1 with an antibody in U937 and Kasumi-1 cells suppressed proliferation, increased apoptosis, down-regulated Bcl-xL and c-Myc expression, up-regulated Bax expression, and had no effect on Survivin. siRNA-mediated down-regulation of CCN1 inhibited the proliferation and colony formation of U937 and Kasumi-1 cells and increased cytarabine-induced apoptosis. Furthermore, CCN1 siRNA reduced MEK and ERK phosphorylation without affecting β-catenin; the CCN1 antibody similarly affected MEK and ERK phosphorylation. These changes in phosphorylation could influence the expression of Bcl-xL, c-Myc and Bax in AML cells.

**Conclusions:**

The data suggested that CCN1 is a tumor promoter in AML that acts through the MEK/ERK pathway to up-regulate c-Myc and Bcl-xL and to down-regulate Bax.

## Background

CCN1 (Cyr61, cysteine-rich 61), a member of the CCN protein family, is an extracellular matrix-associated protein [1]. Domains within the CCN1 protein have binding sites for the various integrins or heparan sulfate proteoglycans (HSPGs) on different cell types, where they activate distinct signaling pathways. CCN1 is essential for development, inflammation, cell adhesion, migration, tumorigenesis and cell survival [2].

CCN1 plays unique roles in different tumor types: it is an oncogenic factor for cancers of the breast [3], prostate [4], and stomach [5] as well as for glioma [6], esophageal squamous cell carcinoma [7], and chondrosarcoma [8]. In contrast, CCN1 is a tumor suppressor in non-small cell lung cancer [9], endometrial adenocarcinoma [10] and melanoma [11]. The role of CCN1 in hepatocellular carcinoma is controversial [12],[13]. However, the role of CCN1 in leukemia is unknown.

Numerous signaling pathways have been implicated in acute myeloid leukemia (AML), including the Wnt/β-catenin [14], RAF/MEK/ERK, and PI3K/AKT [15] pathways. Extracellular signal-regulated kinase (ERK), a member of the MAP kinase family, can be phosphorylated and activated by MEK (mitogen-activated protein kinase/extracellular signal-regulated kinase kinase). Markedly increased MEK or ERK activity has been detected in most cases of AML [16],[17], and ERK activation confers a poor prognosis on AML patients [18]. Inhibition of MEK or ERK suppresses AML cell growth and induces apoptosis [19]-[21].

Here, we show that CCN1 is expressed in two AML cell lines (U937 and Kasumi-1) and in AML bone marrow samples but not in other leukemia cell lines, such as Jurkat (T-ALL), K562 (CML), CEM (T-ALL) or HL-60 (AML) cells. Antibody-mediated CCN1 inhibition increased the apoptosis of both U937 and Kasumi-1 cells; siRNA-mediated CCN1 down-regulation inhibited U937 and Kasumi-1 cell growth and increased cytarabine-induced apoptosis. We demonstrated that CCN1 influences the MEK/ERK pathway in AML cells, potentially regulating c-Myc, Bcl-xL and Bax. These findings suggest that CCN1 is elevated in AML and promotes survival through the MEK/ERK pathway by up-regulating c-Myc and Bcl-xL and by down-regulating Bax. CCN1 could be a diagnostic marker and a therapeutic target for AML.

## Results

### CCN1 expression in leukemia cell lines and AML bone marrow samples

**Figure 1:**
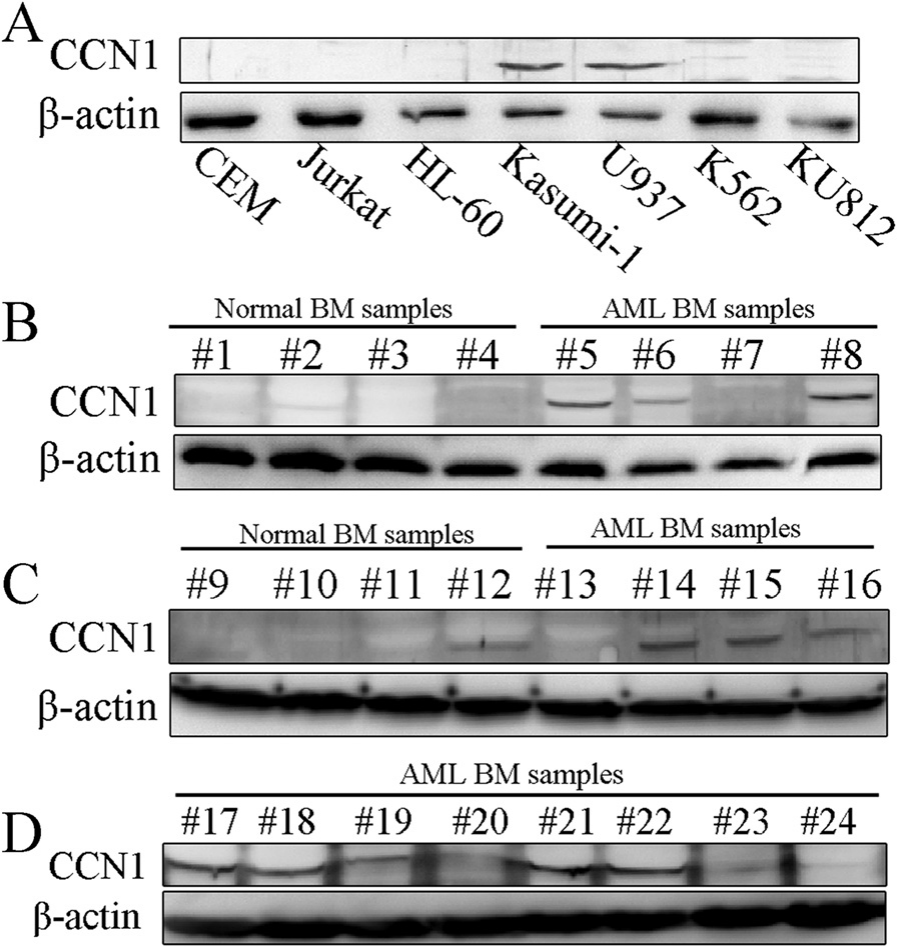
**CCN1 expression in leukemia cell lines and AML bone marrow samples. (A)** Western blotting for CCN1 protein expression in leukemia cell lines. **(B-D)** Western blotting for CCN1 protein expression in AML bone marrow samples and in normal bone marrow samples from healthy donors (#5-8: AML-M1; #13-17: AML-M2; #18-19: AML-M3; #20: AML-M4; #21-24: AML-M5). β-actin served as the loading control. BM, bone marrow.

We analyzed CCN1 expression in seven leukemia cell lines and found the highest levels in two AML lines, U937 and Kasumi-1. CCN1 was undetectable in another AML cell line, HL-60 (Figure 1A). We extracted protein from the mononuclear cells in bone marrow samples and analyzed CCN1 expression to determine whether CCN1 was up-regulated in human AML. There was higher CCN1 expression in the AML samples than in the normal bone marrow samples (Figure 1B-D).

### Blocking CCN1 with an antibody increased apoptosis in U937 and Kasumi-1 cells

CCN1 is a secreted protein that binds to cell-surface receptors to activate a series of signaling pathways [2],[22]. We used an antibody to inhibit CCN1 and examined the role of CCN1 in U937 and Kasumi-1 cell growth.

**Figure 2:**
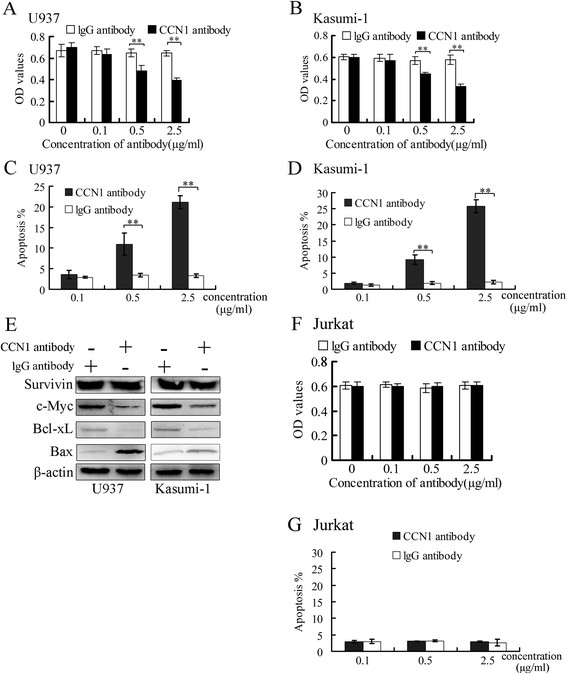
**The CCN1-blocking antibody suppressed U937 and Kasumi-1 cell survival.** U937 and Kasumi-1 cells were treated with the CCN1 antibody or the control antibody (IgG) for 24 hours. **(A, B)** Cell proliferation was measured using a CCK-8 reagent. **(C-D)** The percentage of apoptotic cells was determined using Annexin V-APC/PI staining. **(E)** Western blotting in cells treated with antibodies (0.5 μg/ml) for 24 hours. β-actin served as the loading control. **(F-G)** Jurkat cells were treated with the CCN1 antibody, and proliferation and apoptosis were measured. **p < 0.01.

After a 24-hour incubation with the CCN1 antibody, U937 and Kasumi-1 cell growth was inhibited in a dose-dependent manner compared with a control IgG antibody (Santa Cruz Biotechnology) (Figure 2A, B), and apoptosis was increased (Figure 2C-D). Inhibiting CCN1 activity with the antibody decreased c-Myc and Bcl-xL expression, up-regulated Bax expression and had no effect on Survivin expression (Figure 2E).

Neither the CCN1 antibody nor the control IgG had an effect on the growth or apoptosis of Jurkat cells, a CCN1-negative cell line (Figure 2F-G).

### CCN1 siRNA suppressed U937 and Kasumi-1 cell growth and enhanced the response to cytarabine

We knocked down CCN1 expression with siRNA and constructed stable cell lines (U937/siCCN1 and Kasumi-1/siCCN1) to further study the role of CCN1 in AML cells.

**Figure 3:**
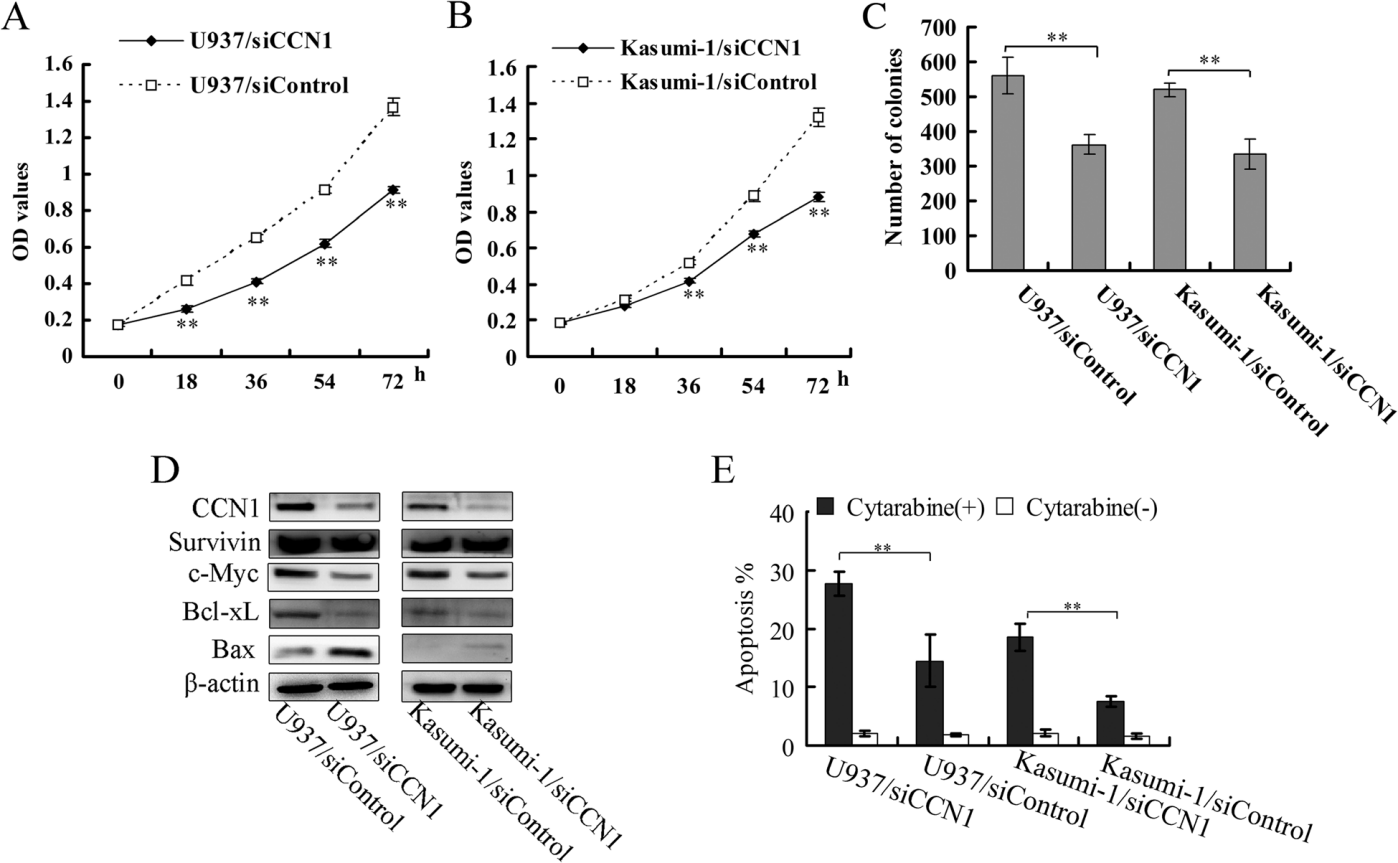
**The influence of CCN1 siRNA on U937 and Kasumi-1 cells. (A, B)** CCK-8 reagent was used to measure AML cell proliferation. h, hour. **(C)** Cells were plated in 6-well plates (2000 cells/well), and the colonies were counted after 7 days. **(D)** Western blotting in AML cells treated with CCN1 siRNA. β-actin served as the loading control. **(E)** The cells were treated with cytarabine (2 μmol/L) for 18 hours, stained with Annexin V-APC/PI and analyzed by flow cytometry. **p < 0.01.

CCN1 siRNA suppressed the proliferation and colony formation of both AML cell lines (Figure 3A-C) to a similar extent as the CCN1-blocking antibody. However, CCN1 siRNA did not increase the percentage of apoptotic cells (data not shown). CCN1 siRNA suppressed c-Myc and Bcl-xL expression and up-regulated the proapoptotic gene Bax (Figure 3D) but had no effect on Survivin expression. AML cells were treated with cytarabine, a common AML therapeutic [23], to determine whether this would render them more susceptible to apoptosis. Cytarabine increased the apoptosis of U937 and Kasumi-1 cells treated with CCN1 siRNA compared with the control cells (Figure 3E).

### The MEK/ERK pathway, but not β-catenin, was involved in CCN1 function in AML cells

**Figure 4:**
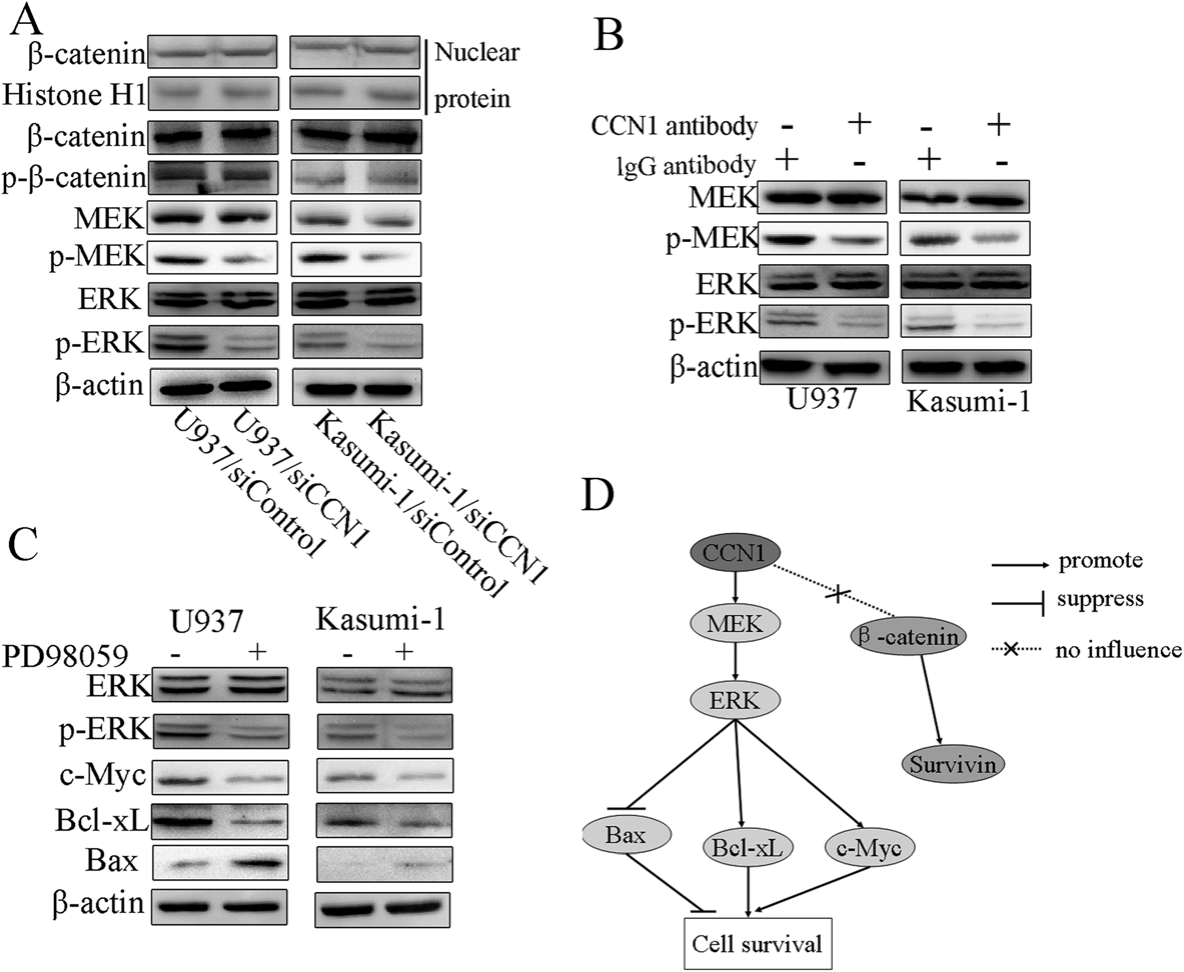
**The MEK/ERK pathway was involved in CCN1 activity in AML. (A)** Western blotting to determine the influence of CCN1 siRNA on β-catenin, MEK and ERK. Histone H1 served as the loading control for nuclear protein. **(B)** Western blotting to illustrate the influence of a 3-hour incubation with the CCN1 antibody on MEK and ERK. **(C)** Western blotting to determine the influence of a MEK/ERK inhibitor (PD98059, 20 μmol/L) on c-Myc, Bcl-xL and Bax. β-actin served as the loading control. **(D)** Schematic of the proteins related to CCN1 in AML cells. p-: phospho-.

CCN1 regulates β-catenin in esophageal squamous carcinoma and lung cancer cells [24],[25]. We previously showed that CCN1 is a direct target of β-catenin signaling in hepatocellular carcinoma (HCC), where it may be important for cancer progression [13]. We therefore determined whether CCN1 functioned in association with β-catenin in AML. However, we found that CCN1 did not affect β-catenin expression or phosphorylation in whole-cell or nuclear extracts (Figure 4A).

Previous studies have shown that CCN1 activates ERK in ovarian carcinoma cells [26] and osteoblasts [27]. Here, we demonstrated that inhibiting CCN1 reduced ERK phosphorylation but not ERK expression (Figure 4A). We next examined the expression of MEK, the major regulator of ERK, and found that CCN1 siRNA inhibited MEK phosphorylation but not its overall expression. The effects of CCN1 on MEK/ERK were also confirmed using the CCN1-neutralizing antibody (Figure 4B). We treated cells with the MEK/ERK inhibitor PD98059 to determine whether MEK and ERK were associated with the CCN1-mediated regulation of Bcl-xL, c-Myc and Bax. Our results indicated that PD98059 had similar effects on these proteins (Figure 4C).

## Discussion

Our results showed that CCN1 was overexpressed in two AML cell lines (U937 and Kasumi-1) and in AML bone marrow samples. However, CCN1 was undetectable in certain AML samples and in one AML cell line (HL-60 cells). These data suggested that CCN1 is not the only factor necessary for AML cell survival. CCN1 may be a tumor promoting factor in AML and could potentially be a diagnostic marker for AML. Our data also showed that CCN1 acts through the MEK/ERK pathway, not the β-catenin/Survivin pathway (Figure 4D).

CCN1 has distinct effects on apoptosis in different cells. CCN1 enhances the Fas-mediated apoptosis of skin fibroblasts [28] and the TRAIL-induced apoptosis of prostate carcinoma cells [29] and induces apoptosis in fibroblasts [30]. In contrast, CCN1 inhibits apoptosis induced by anti-cancer drugs in breast cancer cells [31],[32] and in ovarian carcinoma cells [26],[33],[34]. Our results indicated that a CCN1-blocking antibody increases the apoptosis of two AML cell lines, suggesting that CCN1 inhibits the apoptosis of AML cells. Moreover, CCN1 siRNA inhibited the proliferation and colony formation of AML cells without a concomitant increase in apoptosis. AML cell growth was more robustly inhibited by the CCN1 antibody than by the CCN1 siRNA. Nonetheless, CCN1 siRNA rendered AML cells more sensitive to cytarabine-induced apoptosis.

Previous studies have shown that c-Myc is overexpressed [35] and functions as a proto-oncogene in AML [36],[37]; here, we show that inhibiting CCN1 down-regulated c-Myc protein expression. In AML, Bcl-xL acts as an anti-apoptotic factor, Bax acts as a pro-apoptotic factor [38], and Survivin is regarded as an anti-apoptotic factor [39]. Our finding that inhibiting CCN1 down-regulated Bcl-xL and up-regulated Bax expression without affecting Survivin suggested that CCN1 regulates the proliferation and apoptosis of AML cells through c-Myc, Bcl-xL and Bax.

β-catenin, which is overexpressed in AML, is required for the self-renewal of AML stem cells [40]-[43], and inhibiting β-catenin suppresses the proliferation of AML cells [43],[44]. β-catenin is regulated by CCN1 in esophageal squamous carcinoma and lung cancer [24],[25]. We investigated whether CCN1 function in AML is related to β-catenin, but our finding that CCN1 inhibition had no effect on β-catenin or its target gene Survivin [45] does not support this relationship.

As mentioned in the introduction, ERK is activated in AML and is a potential target for AML therapy. ERK is regulated by CCN1 in breast cancer and during osteoblast differentiation [27],[32]. We found that MEK/ERK was regulated by CCN1 in AML cells, and MEK/ERK had the same effect as CCN1 on the regulation of Bcl-xL, c-Myc and Bax. Our study suggests that CCN1 regulates Bcl-xL, c-Myc, and Bax and acts as a tumor promoter through the MEK/ERK pathway. MEK/ERK inhibition enhances the response of AML cells to several chemical treatments [46]-[50], including cytarabine [51]; therefore, we hypothesize that inhibiting CCN1 increases the cytarabine-induced apoptosis of AML cells by down-regulating the MEK/ERK signaling pathway.

## Conclusions

We demonstrated that CCN1 is up-regulated in AML bone marrow samples and that CCN1 promotes AML cell growth. These functions of CCN1 involve the activation of the MEK/ERK pathway, but not the β-catenin/Survivin pathway, with the consequent up-regulation of c-Myc and Bcl-xL and down-regulation of Bax. Our study furthers our understanding of the role of CCN1 in AML and provides a potential alternative therapeutic target and/or diagnostic marker for AML.

## Methods

### Cell lines, primary samples and reagents

The U937 and Kasumi-1 AML cell lines, the Jurkat and CEM T-ALL cell lines, and the K562 CML cell line were maintained in RPMI-1640 medium (Hyclone) with 10% fetal bovine serum (FBS, Gibco). The KU812 CML cell line and the HL-60 AML cell line were maintained in IMDM medium (Hyclone) with 20% FBS, and HEK293 cells were maintained in DMEM (Hyclone) with 10% FBS.

Bone marrow samples from sixteen newly diagnosed and untreated AML patients and from 8 healthy donors were obtained from Chongqing Xinqiao Hospital. The patients and healthy donors provided informed consent, and the study was approved by the Ethical Committee of Third Military Medical University. Mononuclear cells were isolated by density gradient centrifugation using Ficoll-Hypaque (Tianjin TBD, China).

The ERK inhibitor PD98059 (Sigma-Aldrich) was dissolved in DMSO (Sigma-Aldrich). Cytarabine was purchased from Sigma-Aldrich.

### RNA interference

The CCN1 siRNA targeted the sequence 5′-GGGAAAGTTTCCAGCCCAA-3′, and the control sequence did not target any genes. The sequences were cloned into the pSEB-hus plasmid, and this plasmid was transfected into HEK293 cells along with the pCL-Ampho plasmid to package retrovirus. Retroviral supernatants were used to infect U937 and Kasumi-1 cells. The infected cells were maintained under selection using Blasticidin S (Invitrogen, USA). The cells infected with CCN1 siRNA were termed U937/siCCN1 or Kasumi-1/siCCN1; the control cells were denoted U937/siControl or Kasumi-1/siControl.

### Apoptosis and proliferation assays

Apoptosis assay: Cells were washed 3 times with ice-cold PBS and stained with Annexin V-APC (Keygentec, China) and propidium iodide (PI, Sigma-Aldrich). Apoptotic cells were identified by flow cytometry (BD Biosciences) based on positive staining for Annexin V-APC and PI negativity.

Proliferation assay: Ten microliters of CCK-8 reagent (Dojindo, Japan) was added to the wells of 96-well plates in which cells were cultured (100 μl/well). After a 90-minute incubation at 37°C, the OD values were measured using a Bio-Rad Microplate Reader.

### Colony formation assay

Cells (2 × 10^3^) were plated in 6-well plates in a methylcellulose (Sigma-Aldrich) semi-solid medium (final concentration, 1%). After seven days, colonies containing more than 40 cells were counted.

### Western blotting

Western blotting was performed as described previously [13]. The following antibodies were used: CCN1 antibody (Abcam), β-actin antibody (Santa Cruz Biotechnology), Bcl-xL antibody (Cell Signaling Technology), c-Myc antibody (Cell Signaling Technology), Bax antibody (Santa Cruz Biotechnology), MEK antibody (Cell Signaling Technology), phospho-MEK antibody (Ser217/221, Cell Signaling Technology), ERK antibody (Cell Signaling Technology), phospho-ERK antibody (Thr202/Tyr204, Cell Signaling Technology), β-catenin antibody (Cell Signaling Technology), phospho-β-catenin antibody (Ser33/Ser37/Thr41, Cell Signaling Technology), and Survivin antibody (Cell Signaling Technology).

Gel electrophoresis and transfer as well as the chemiluminescence detection were conducted using the Bio-Rad Laboratories system.

Nuclear protein was extracted using a nuclear protein extraction kit (Beyotime, China), and an anti-Histone H1 antibody (Santa Cruz Biotechnology) was used as the loading control.

### Statistical analysis

The experiments were conducted three times, and the data are presented as the means ± SD. Significance was determined by Student’s t-test using SPSS software. p < 0.05 was considered to be statistically significant.

## Competing interests

The authors declare that they have no competing interests.

## Authors’ contribution

Chang-Chun Niu, Wei-Ke Si: the conception and design of the study; Chang-Chun Niu, Chen Zhao, Xiao-Li Zhang, Jing Pan, Chen Zhao, Jing Pan: acquisition of data, or analysis and interpretation of data; Chang-Chun Niu, Zhong Yang, Wei-Ke Si : drafting the article or revising it critically for important intellectual content. All authors read and approved the final manuscript.
